# Supplementary material for: Evaluation of a commercial ELISA as alternative to plaque reduction neutralization test to detect neutralizing antibodies against SARS-CoV-2
Source: Sci Rep. 2022 Mar 3;12:3549. doi: 10.1038/s41598-022-07597-3 (PMC8894493; doi:10.1038/s41598-022-07597-3)
Supplement: Supplementary file 1 — Supplementary Information. [file 41598_2022_7597_MOESM1_ESM.pdf]

Evaluation of a commercial ELISA as alternative to plaque reduction neutralization test to detect neutralizing antibodies against SARS-CoV-2

Natalie Hofmann[1,#], Marica Grossegesse[1,#,*], Markus Neumann[1,2], Lars Schaade[3] and Andreas Nitsche[1]

[1]Highly Pathogenic Viruses, Centre for Biological Threats and Special Pathogens, WHO Reference Laboratory for SARS-CoV-2 and WHO Collaborating Centre for Emerging Infections and Biological Threats, Robert Koch Institute, Berlin, Germany.

[2]Public Health, Infection Control and Rescue Services (Unit 650), Ministry of Economics, Employment and Health, Schwerin, Germany

[3]Centre for Biological Threats and Special Pathogens, WHO Reference Laboratory for SARS-CoV-2 and WHO Collaborating Centre for Emerging Infections and Biological Threats, Robert Koch Institute, Berlin, Germany.

[#]The authors contributed equally

[*]Corresponding author

Content

Table S1. Evaluation of the effect of different cutoffs and equivocal areas on the diagnostic performance of the GenScript sVNT……………………………………………………………………………………………………**2**

Table S2. Details of tested ELISAs and sample subsets used for the comparative evaluation……………..**2**

Table S3. Comparison of the diagnostic performance of GenScript sVNT to other commercial ELISAs.**3**

Figure S1. Receiver operating characteristic (ROC) curve for the GenScript sVNT……………………………..**3**

Table S1. Evaluation of the effect of different cutoffs and equivocal areas on the diagnostic performance of the GenScript sVNT

		Number of samples (classified by GenScript sVNT)			Counting indeterminate as positive		Counting indeterminate as negative	
	PRNT	Positive	Indeter-minate	Negative	Sensitivity	Specificity	Sensitivity	Specificity
No equivocal area (cut-off at 30%)	pos	188	0	58	76.42%	95.92%	76.42%	95.92%
	neg	10	0	235				
Equivocal area from 10-40%	pos	159	71	16	93.50%	57.96%	64.63%	97.55%
	neg	6	97	142				
Equivocal area from 15-35%	pos	177	52	17	93.09%	73.47%	71.95%	97.55%
	neg	6	59	180				
Equivocal area from 20-30%	pos	188	32	26	89.43%	87.35%	76.42%	95.92%
	neg	10	21	214				

Table S2. Details of tested ELISAs and sample subsets used for the comparative evaluation.

**ELISA**	**Type**	**Qualitative / Quantitative**	**Target**	**No of samples tested in comparison to PRNT**
Detection of neutralizing Antibodies				
GenScript cPass SARS-CoV-2 Neutralization Ab detection kit	Competitive	Semiquantitative	RBD	491 convalescent<br>112 vaccinated
Wantai NAbs	Competitive	Qualitative	S	111
EuroImmun SARS-CoV-2 NeutraLISA	Competitive	Semiquantitative	RBD	111
Detection of binding Antibodies				
Euroimmun S1 IgG ELISA	Indirect	Semiquantitative	S1	298
Euroimmun NCP IgG ELISA	Indirect	Semiquantitative	NCP	205
Wantai complete Ab ELISA	Sandwich	Qualitative	RBD	388

Table S3. Comparison of the diagnostic performance of GenScript sVNT to other commercial ELISAs, using PRNT as gold standard

		Commercial ELISA (Euroimmun S1 IgG / Euroimmun NCP IgG / Wantai complete Ab, respectively)					GenScript sVNT (using in-house validated cutoffs)				
	PRNT	Pos	IND[1]	Neg	Sensitivity[2]	Specificity[2]	Pos	IND[1]	Neg	Sensitivity[2]	Specificity[2]
Full sample set	pos						185	52	17	72.8 - 93.3%	73.8 - 97.6%
	neg						6	60	180		
Euroimmun S1 IgG sample set	pos	113	14	10	82.5 - 92.7%	64.0 – 78.9%	105	23	9	76.8 - 93.%	76.4 - 98.8%
	neg	34	24	103			2	36	123		
Euroimmun NCP IgG sample set	pos	103	12	5	85.8 - 95.8%	85.9 - 90.6%	98	13	9	81.7 - 92.5%	75.3 - 98.8%
	neg	8	4	73			1	20	64		
Wantai complete Ab sample set	pos	178	0	4	97.8%	71.8%	122	43	17	67.0 – 90.7%	74.8 - 97.6%
	neg	58	0	148			5	47	154		

[1] IND: Indeterminate

[2] Ranges depend on whether indeterminate samples were counted as positive or negative

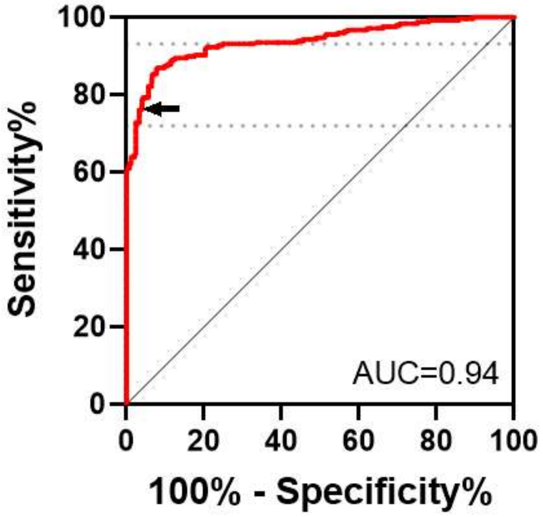

Figure S1. Receiver operating characteristic (ROC) curve for the GenScript sVNT.
Arrow: manufacturer-recommended cutoff (30% inhibition). Grey dashed lines: in-house defined equivocal area (15-35% inhibition).
